# A Replicating Single-Cycle Adenovirus Vaccine Effective against *Clostridium difficile*

**DOI:** 10.3390/vaccines8030470

**Published:** 2020-08-22

**Authors:** William E. Matchett, Stephanie Anguiano-Zarate, Goda Baddage Rakitha Malewana, Haley Mudrick, Melissa Weldy, Clayton Evert, Alexander Khoruts, Michael Sadowsky, Michael A. Barry

**Affiliations:** 1 Virology and Gene Therapy (VGT) Graduate Program, Mayo Clinic, Rochester, MN 55905, USA; matc0017@umn.edu; 2 Clinical and Translational Science (CTS) Graduate Program, Mayo Clinic, Rochester, MN 55905, USA; anguiano.zarate.stephanie@gmail.com; 3 Mayo Summer Undergraduate Research Fellow (SURF), Mayo Clinic, Rochester, MN 55905, USA; rakitha.malewana@duke.edu; 4 Molecular Pharmacology and Experimental Therapeutics (MPET) Graduate Program, Mayo Clinic, Rochester, MN 55905, USA; mudrick.haley@mayo.edu; 5 Inflammatory Bowel Program, Division of Gastroenterology, Hepatology and Nutrition, University of Minnesota, Minneapolis, MN 55454, USA; weld0002@umn.edu (M.W.); ever0123@umn.edu (C.E.); khoru001@umn.edu (A.K.); sadowsky@umn.edu (M.S.); 6 BioTechnology Institute, University of Minnesota, St Paul, MN 55108, USA; 7 Department of Surgery, University of Minnesota, Minneapolis, MN 55455, USA; 8 Department of Soil, Water, and Climate Department of Plant and Microbial Biology, University of Minnesota, University of Minnesota, St Paul, MN 55108, USA; 9 Department of Internal Medicine, Division of Infectious Diseases, Mayo Clinic, Rochester, MN 55905, USA; 10 Department of Immunology, Mayo Clinic, Rochester, MN 55905, USA; 11 Department of Molecular Medicine, Mayo Clinic, Rochester, MN 55905, USA

**Keywords:** Clostridium difficile, adenovirus, single-cycle, vaccine, animal models

## Abstract

*Clostridium difficile* causes nearly 500,000 infections and nearly 30,000 deaths each year in the U.S., which is estimated to cost $4.8 billion. *C. difficile* infection (CDI) arises from bacteria colonizing the large intestine and releasing two toxins, toxin A (TcdA) and toxin B (TcdB). Generating humoral immunity against *C. difficile*’s toxins provides protection against primary infection and recurrence. Thus, a vaccine may offer the best opportunity for sustained, long-term protection. We developed a novel single-cycle adenovirus (SC-Ad) vaccine against *C. difficile* expressing the receptor-binding domains from TcdA and TcdB. The single immunization of mice generated sustained toxin-binding antibody responses and protected them from lethal toxin challenge for up to 38 weeks. Immunized Syrian hamsters produced significant toxin-neutralizing antibodies that increased over 36 weeks. Single intramuscular immunization provided complete protection against lethal BI/NAP1/027 spore challenge 45 weeks later. These data suggest that this replicating vaccine may prove useful against CDI in humans.

## 1. Introduction

*Clostridium difficle* (recently renamed *Clostridioides difficile* [1]) is an anaerobic, Gram-positive, spore-forming bacterium that has emerged as a significant enteric pathogen (reviewed in [2]). Each year in the United States, *C. difficile* is estimated to cause 500,000 infections and results in about 29,000 deaths [3]. *C. difficile* infection (CDI) occurs most after antibiotic therapy. Antibiotics disrupt healthy intestinal microbiota enabling pathologic *C. difficile* overgrowth [4]. CDI can cause symptoms ranging from frequent watery diarrhea to bowel perforation and death [5]. 

One approach to mitigate CDI is through prevention strategies, such as the use of a vaccine for individuals with risk factors for disease, which include advanced age, duration of stay during hospitalization, and exposure to antimicrobial agents [6]. *C. difficile* vaccines generally target the pathogenic, secreted toxins, toxin A (TcdA) and/or toxin B (TcdB), which are expressed by most clinical isolates [7]. In a hamster model, targeting both toxins with antibodies reduces CDI incidence more than targeting either toxin alone [8,9,10]. Protection against CDI in humans and in hamsters correlates with antibody responses to TcdA and TcdB [8,11]. This suggests that targeting both virulence factors in a vaccine would be the optimal strategy.

Both toxins bind enterocytes in the colon and release a glucosyl transferase into cells that disrupts the cytoskeleton, resulting in cell death [12]. The damage may lead to fluid accumulation, inflammation in the colon, and uncontrolled diarrhea. Both toxins have a common type ABCD domain structure; each toxin possesses glucosyltransferase catalytic A domain on their n-terminus and a “B” receptor binding domain (RBD) on their c-terminus [13]. These RBDs bind receptors on the surfaces of epithelial cells in the digestive tract [14]. Vaccines using recombinant antigens derived from the RBDs have generated strong neutralizing antibodies in animal models and human trials [15,16].

One challenge in developing a vaccine against the toxins is avoiding the cytotoxicity intrinsic to the enzymatic A domain. Therefore, many efforts have involved the use of inactivated toxins (toxoids) [17]. A toxoid TcdA and TcdB protein vaccine is currently being tested in clinical trials [18]. This vaccine protected hamsters in preclinical studies and is showing promise in clinical trials [16,19]. Since antibodies against the RBDs of the toxins are capable of neutralizing the toxins in vitro and in vivo [20], other vaccines have focused on generating antibody responses against the RBDs of TcdA and B. Despite their efficacy in animal models, these protein vaccines must be delivered at least three times to generate strong antibody responses [17]. Even with multiple rounds of immunization, one of the candidate toxoid vaccines still failed to meet clinical trial efficacy goals [21].

Given the need for new interventions, we describe here the development of a novel gene-based vaccine against *C. difficile*. As in previous studies, we used a single-cycle adenovirus (SC-Ad) vector as a replicating gene-based vaccine platform [22,23,24,25,26]. Unlike replication-defective plasmid or replication-defective Ad vaccines, the SC-Ad vectors replicate antigen genes thousands of times to amplify immune responses. Yet, unlike replication-competent Ads, SC-Ad cannot cause adenovirus infections. Thus, SC-Ad vectors are safer than replication-competent Ads and more immunogenic than replication-defective viruses. We engineered the low seroprevalence SC-Ad6 to express RBDs from *C. difficile’s* two toxins. We have evaluated its ability to generate neutralizing antibody responses and have tested its ability to protect against *C. difficile* toxins.

## 2. Materials and Methods 

### 2.1. Cell Culture 

The A549 and Vero cells were purchased from the American Type Culture Collection (Manassas, VA, USA). The 293-IIIA cells were generated as described in [19]. All cells were maintained at 37 °C in Dulbecco’s Modified Eagle Medium (DMEM) supplemented with 10% heat-inactivated fetal bovine serum (HI-FBS; Hyclone, South Logan, UT, USA) and penicillin/streptomycin at 100 U/mL (Invitrogen, Waltham, MA). 

### 2.2. Single-Cycle Adenoviruses

A codon-optimized cDNA encoding a novel fusion of the receptor binding domains (RBDs) of *C. difficile* toxins A and B was synthesized by Genscript (Piscataway, NJ). This cDNA contains the secretory leader sequence from alpha-1 antitrypsin (AAT) to facilitate secretion of the protein. This vector carries a mammalian codon-optimized cDNA that expresses a fusion protein consisting of a secretory leader and the RBDs of TcdA and B separated by two furin cleavage sites to allow the two RBD to be seperated. The toxin RBDs were derived from the strain VPI 10,463 (toxinotype 0) with glutamine for the asparagine substitutions in putative n-linked glycosylation sites, which has been shown to enhance neutralizing antibody (nAb) responses [27]. This resulted in a total of eight substitutions in the TcdA RBD and three alterations within the TcdB RBD sequences. This cDNA was inserted into the shuttle plasmids pAd6-NdePfl and was recombined into SC-Ad6 as described in [22,24,28] to generate SC-Ad6-C.diff depicted in Figure S1. Control SC-Ad6 viruses expressing GFP-Luciferase or *Campylobacter jejuni* cell-binding factor PEB1 were also used. Viruses were rescued, amplified, and purified as described previously [22,24,28]. Virus comparisons were based on virus particles (vp) [29]. 

### 2.3. Western Blotting

The A549 cells were infected with SC-Ad6-TcdA/B or SC-Ad-GL, which expresses GFP-Luciferase, with 10^4^ vp/cell. Twenty-four h after infection, the media was replaced with serum-free DMEM. Twenty-four h later, this media was collected and concentrated using Amicon Ultra-15 30k (Millipore, Burlington, MA, USA). Cells were harvested with lysis buffer and protease inhibitors as in [30]. Media concentrates and cell lysates were analyzed by Western blot (Figure S2) using antibodies against *C. difficile* toxin A or B (List Biological Labs, Inc., Campbell, CA, USA) followed by a goat anti-chicken horseradish peroxidase secondary antibody (Invitrogen). SuperSignal West Dura (Thermo Fisher Scientific, Waltham, MA, USA) was added and blots were imaged on an In Vivo F Station (Kodak, New Haven, CT, USA). 

### 2.4. Animals

Male and female outbred CD-1 mice (Charles River Laboratories, Wilmington, MA; USA) and female Golden Syrian hamsters (Envigo, Indianapolis, Indiana) were housed in the Mayo Clinic Animal Facility. All animal handling and experiments were carried out according to the provisions of the Animal Welfare Act, PHS Animal Welfare Policy, the principles of the NIH Guide for the Care and Use of Laboratory Animals, and the policies and procedures of the Institutional Animal Care and Use Committee at Mayo Clinic. 

### 2.5. Immunizations and Sample Collection

Mice were anesthetized with isoflurane and immunized via the intramuscular (i.m.) route with 10^10^ vp of the indicated vaccine. Hamsters were anesthetized with isoflurane and immunized intramuscularly (i.m.) or intranasally (i.n.) with 10^11^ vp of the SC-Ad vaccines. Hamsters were also immunized with a mixture containing 10 ug toxoid A and 10 ug of B (Native Antigen Company, Oxford, GB) suspended with aluminum phosphate (25 μg) (Sigma Aldrich, St. Louis, MO, USA) or aluminum phosphate (25 μg) alone. In mice, serum was collected from the facial vein at the indicated time points. At various time points, hamsters were anesthetized, and blood was collected. 

### 2.6. Enzyme-Linked Immunosorbent Assay (ELISA)

Immulon 4 HBX plates (Thermo Fisher Scientific) were coated with 100 ng/well of either *C. difficile* A or B toxoids (List Biological Labs, Inc.) in 1X phosphate-buffered saline (PBS) overnight. Wells were washed and blocked with 5% milk in Tris-buffered saline with 0.1% Tween 20 (TBST) at room temperature (RT) for 2 h. After washing with TBST, half-log dilutions of each serum sample were plated in triplicate and incubated for 3 h at RT. Wells were washed and goat anti-mouse IgG-horseradish peroxidase (Thermo Fisher Scientific) was added to each well. Plates were incubated for 2 h at RT. Wells were washed and 1 step Ultra TMB ELISA (Thermo Fisher Scientific) was added to each well. When color developed, 2M H_2_SO_4_ was added. OD450 was determined with a BioTek Synergy H1 Hybrid Multi-Mode Reader. Reciprocal titers were statistically defined based on 95% confidence interval defined previously [31].

### 2.7. Cytotoxicity and Neutralization Assays

Cytotoxicity of *C. difficile* toxin A and toxin B were determined on Vero cells using the method described by Robert Donald [19]. Vero cells were used instead of IMR-90 as they have similar sensitivity to Toxin B compared to IMR-90 cells, but they have an increased sensitivity to toxin A [32]. Vero cells were plated at 10^4^ cells per well in a 96-well plate. *C. difficile* toxin A or toxin B (List Biological Laboratories, Inc., Campbell, CA, USA) was serially diluted in DMEM supplemented with 10% HI-FBS and added to cells 24 h after plating. Three days later, cell viability was determined using the bioluminescent CellTiter-Glo reagent (Promega, Madison, WI). By fitting the data with a four-parameter equation, the half-maximal effective concentration (EC50) was determined to be the amount of toxin causing 50% reduction in luminescence. Toxin neutralization was determined using serial dilutions of mouse or hamster serum mixed with eight times the EC50 value determined in the cytotoxicity assay [19]. The mixtures were incubated at 37 °C for 90 min in a humidified incubator (5% CO2) before being added to Vero cells on 96 well-plates. Three days later, cell viability was determined with Celltiter-Glo. A four-parameter regression response was fitted to the luciferase relative light units (RLU) values derived from the serum dilutions. nAb titers were expressed as the derived sample dilution that exhibited a 50% reduction in cytotoxicity. If a serum titration failed to generate 50% inhibition within the range of concentrations tested, a titer value of ½ of the highest serum concentration tested was ascribed to it.

### 2.8. Challenge with Recombinant C. difficile Toxin A in Mice

As in previous studies, immunized mice were challenged intraperitoneally with 300 ng of *C. difficile* toxin A (List Biological Laboratories, Inc., Campbell, CA, USA) [33,34]. Following toxin challenge, mice were monitored every 3 h for the first 30 h, followed by monitoring at 6-h intervals for 72 h, and then every 12 h from day 3 through day 7 (168 h). Mice were monitored for clinical signs defined in Qiu, H. et al. [35]. Briefly, animals’ symptoms were scored as normal, lethargic, abnormal, or moribund. Moribund animals were euthanized immediately and recorded. The survival rate was determined for each treatment group.

### 2.9. Hematology and Clinical Chemistry in Hamsters

Blood was collected for clinical chemistry analysis (200 μL into lithium heparin tubes; Greiner Bio-One) and for complete blood count (CBC, 100 μL in K_2_ EDTA tubes; Greiner Bio-One, Monroe, NC, USA). Blood chemistry was analyzed with a Piccolo Xpress Analyzer (Abaxis) and CBCs were determined with VetScan HM5 hematology analyzer (Abaxis, Mountain View, CA, USA). Analyte parameters for the two tests are shown in Tables S1 and S2.

### 2.10. Challenge with C. difficile Spores in Hamsters

Prior to challenge, hamsters were housed individually in ventilated cages. In the low-dose challenge, hamsters were sensitized for infection using a clindamycin phosphate (Sigma-Aldrich) antibiotic solution (30 mg/kg of body weight) delivered orogastrically via a feeding needle. Five days later, the hamsters were challenged orogastrically with 200 spores from the *C. difficle* strain UK1 that had been prepared as previously described [36]. Since low-dose spore challenge did not induce symptoms in hamsters in our hands, a high-dose challenge with modified clindamycin administration was adapted from Anosova [37]. In this challenge, hamsters were sensitized with clindamycin antibiotic solution (10 mg/kg of body weight) by the intraperitoneal route. Then, the hamsters were challenged 24 h later orogastrically with 10^4^ UK1 spores. In both the high and low-dose challenge, the hamsters were monitored 4 times per day following infection by assessing them individually for several parameters, including presence and severity of wet tail, loose feces, diarrhea, weight loss, activity level, starey coat, sunken eyes, hunched posture, and response to stimulus. A scoring system, based on the severity of changes observed (ranging from 0 to 3 for each parameter) [38], was used to quantify the condition of the animals. Animals were euthanized and considered to have succumbed to disease when they either reached a score ≥ 15, were moribund, or suffered weight loss in excess of 20%.

### 2.11. Statistical Analysis

Prism 8 Graphical software was used for all statistical analyses. Comparisons between two groups were tested for significance by the Mann–Whitney U test, multiple comparisons were made by Dunn’s test, and survival was tested by Log-Rank analysis. For time course analysis of antibody responses, Dunn’s tests were performed individually to compare samples collected on the same date.

## 3. Results

**Figure 1:**
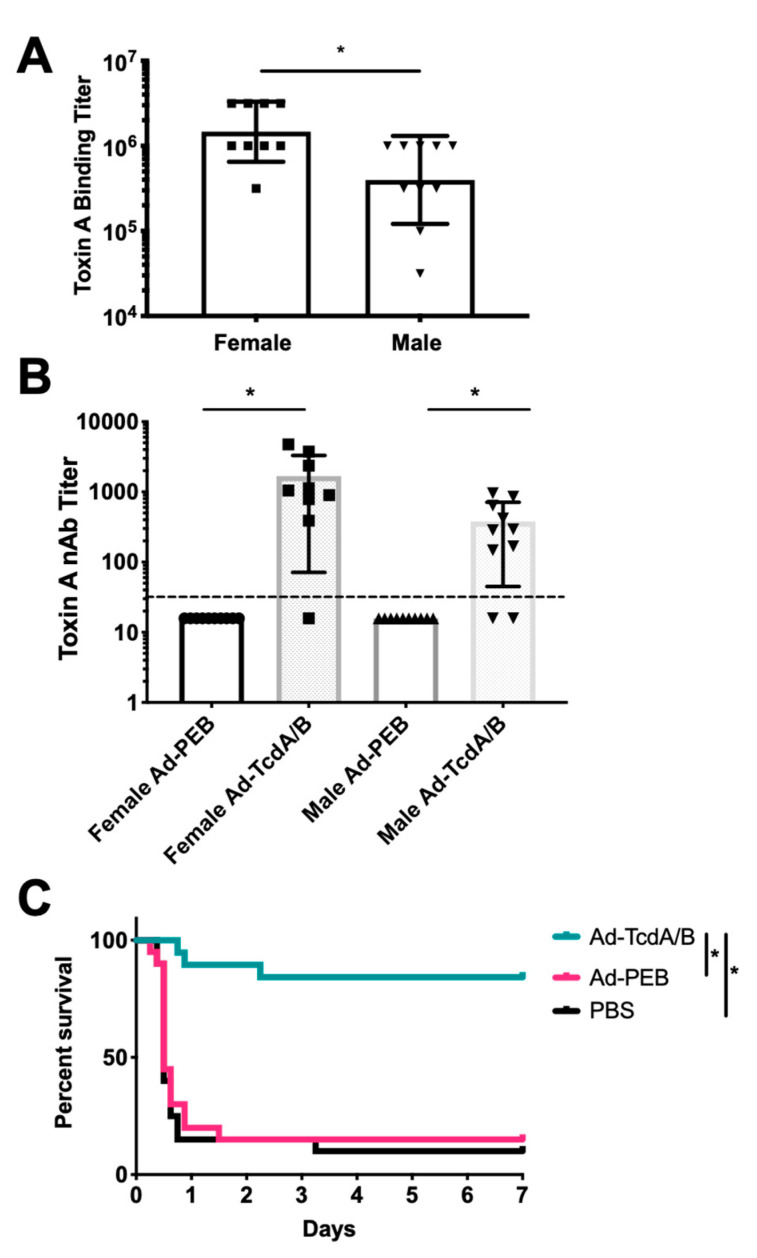
Serum antibody responses in immunized CD-1 mice and toxin A challenge. Male and female CD-1 mice (n = 10, per group) were vaccinated intramuscular (i.m.) with 1 × 10^10^ virus particles of SC-Ad6-PEB1, SC-Ad6-TcdA/B, or phosphate-buffered saline (PBS). Serum collected at week 6 was assayed by ELISA and neutralization assay. (**A**) Serum endpoint titers were determined at week 6 for male and female mice. Columns show geometric mean; bars show standard deviation; (* *p* < 0.01). (**B**) Neutralizing titers for Toxin A. Columns show mean; bars show standard deviation; dotted line indicates the limit of detection; (* *p* < 0.05). (**C**) Combined male and female survival curve following Toxin A challenge at week 8 after single immunization (* *p* < 0.01).

Groups of male and female outbred CD-1 mice were immunized via i.m. injection a single time with PBS, 10^10^ virus particles of SC-Ad6-TcdA/B, or a negative control vector SC-Ad6-PEB1 that expresses a mismatched protein from another bacterium, Campylobacter jejuni (n = 10 per group). Single immunization generated significant antibodies against TcdA in the majority of the SC-Ad6-TcdA/B vaccinated animals within 6 weeks (Figure 1A,B). Serum endpoint titers at week 6 were significantly higher in female mice immunized with SC-Ad-TcdA/B than in males (Figure 1A). Neutralizing titers aginst TcdA were significantly higher in the female (160-fold increase) and male (40-fold increase) groups compared to sex-matched controls. (Figure 1B). Both sexes of animals generated significant antibody responses. Female binding titers were significantly higher than male titers. A similar pattern was observed in the TcdA nAb titers from the two sexes, but these differences were not significant. When these were compared to animals immunized with SC-Ad6-PEB1, those receiving SC-Ad6-TcdA/B had nAb titers that were significantly higher than their sex-matched controls.

Eight weeks after a single immunization, the groups of 10 males and 10 female mice were challenged with 300 ng (6 × LD50) of purified TcdA from a ribotype 087 and toxinotype 0 strain similar to VPI 10,463 antigens in the SC-Ad vaccine as in Seregin et al. [34]. Eighteen of 20 PBS and PEB1 mice succumbed to the toxin within 24 h of challenge (Figure 1C). One additional PBS mouse met sacrifice criteria 3 days later. In marked contrast, 17 out of the 20 SC-Ad6-TcdA/B vaccinated mice survived the challenge with 2 males and 1 female not surviving the challenge. Comparison of the survival curves demonstrated that SC-Ad6-TcdA/B vaccinated animals survived significantly better than PBS or PEB1 control animals. Interestingly, the 2 males and 1 female SC-Ad6-TcdA/B vaccinated mice that did not survive had lower TcdA binding titers and a complete absence of nAbs (Figure 1A,B).

**Figure 2:**
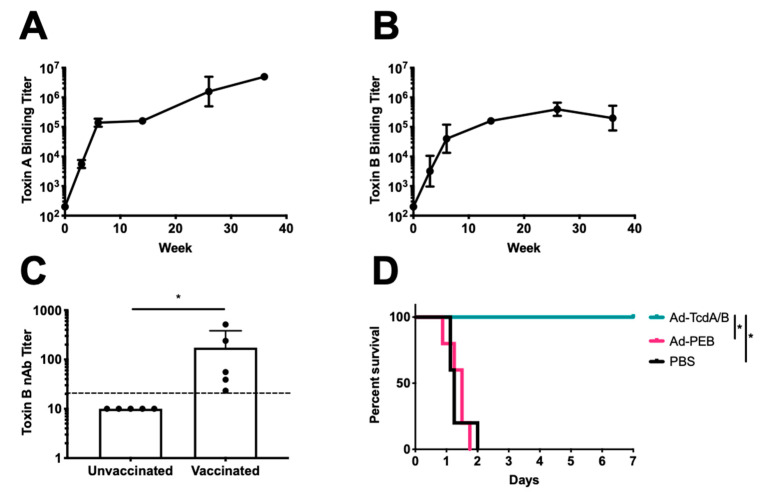
SC-Ad6-TcdA/B provides protection against lethal challenge long after single immunization. Female CD-1 mice (n = 5) were vaccinated i.m. with 1×10^10^ virus particles of SC-Ad6-PEB1, SC-Ad6-TcdA/B, or PBS. Serum collected at weeks 3, 6, 14, 26, and 36 were titrated to determine binding endpoint titers for (**A**) toxin A and (**B**) toxin B. In both panels, points show geometric mean and bars show standard deviation. (**C**) Mean neutralizing titers for toxin B were significantly higher in the SC-Ad-TcdA/B than SC-Ad-PEB1 immunized animals. Columns show mean; bars show standard deviation; the dotted line indicates the limit of detection; (* *p* < 0.05). (**D**) Survival curve for SC-Ad-TcdA/B vaccinated mice challenged with toxin A shows significant protection compared to PBS or PEB1 control animals (* *p* < 0.01).

A second set of female CD-1 mice were immunized i.m. a single time with 10^10^ virus particles of SC-Ad6-TcdA/B, SC-Ad6-PEB1, or PBS (n = 5 per group). This single vaccination of SC-Ad6-TcdA/B generated strong antibody responses with reciprocal endpoint binding titers for TcdA and TcdB over 26 weeks that climbed above 100,000 (Figure 2A,B). At week 26, we measured the TcdB neurtralizing titers and found that the vaccinated animals’ titers were 17-fold greater than controls (Figure 2C). At week 38, the mice were challenged with 300 ng (6 × LD50) of TcdA. All PBS and SC-Ad-PEB1 animals succumbed to the toxin within 48 h (Figure 2D). All animals in the SC-Ad-TcdA/B vaccine group survived.

Groups of 10 Syrian hamsters were immunized a single time with 10^11^ virus particles of SC-Ad6-TcdA/B by the i.n. or i.m. routes. Control animals received i.n. PBS. Blood was collected 3 days after immunization for clinical chemistry. These revealed no significant differences in blood chemistry (Figure S3). Furthermore, the values of these analytes were within the reference ranges [39]. 

**Figure 3:**
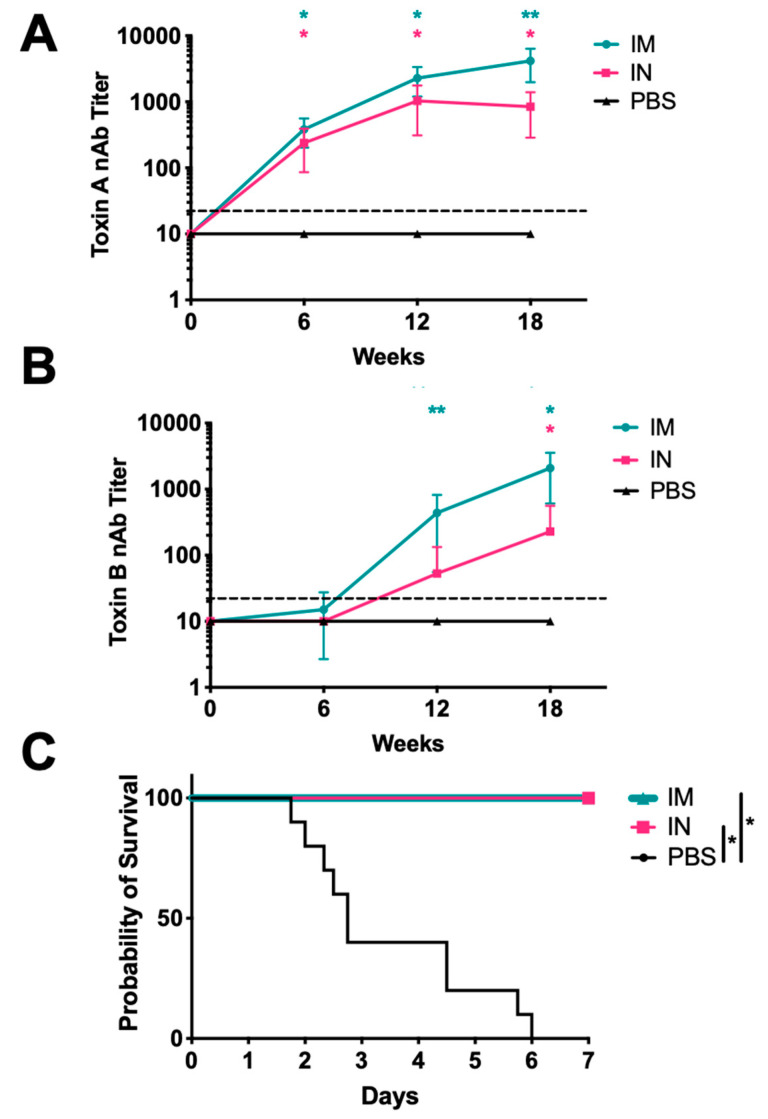
Serum-neutralizing antibody responses in immunized Syrian hamsters and protection from lethal spore challenge. Female Syrian hamsters (n = 10) were vaccinated i.n. or i.m. with 1 × 10^11^ virus particles of SC-Ad6-TcdA/B, or i.n. with PBS. Serum collected at 6, 12, and 18 weeks after immunization were assayed to determine mean neutralizing titers for (**A**) toxin A and (**B**) toxin B. In both panels, points show the mean titer, bars show standard deviation, and the dotted line indicates the limit of detection (*Adjusted *p* < 0.05 compared to control, **Adjusted *p* < 0.05 compared to control and i.n. route). (**C**) Survival curve for SC-Ad-TcdA/B vaccinated animals challenged with UK1 spores shows significant protection compared to PBS control animals (* *p* < 0.01).

Single immunization of the hamsters with SC-Ad6-TcdA/B generated significant serum nAbs against TcdA regardless of route that increased over time (Figure 3A). While i.m. immunization produced higher mean nAbs against toxin A than the i.n. group, these were not significantly different until week 18. At this time point, TcdA nAb titers were 400-fold greater in the i.m. group than the control group), and 80-fold greater in the i.n. group. TcdB antibodies were detectable by ELISA at week 6 (data not shown), but they took longer to develop (Figure 3B). At week 18, all i.m. and i.n. immunized animals had significant TcdB nAbs, which were 200-fold and 20-fold greater than the control, respectively.

CDI can be induced in Syrian hamsters when sensitized with clindamycin. This mimics the fecal-oral route of transmission and produces symptoms similar to those observed in patients with CDI [40]. Various toxin isoforms have been identified within clinical isolates of *C. difficile*. Recent studies report that the BI/NAP1/027 strain of *C. difficile* is the most prevalent cause of CDI in North America [41,42]. Given its clinical relevance and expression of heterologous isoforms of the toxins, we tested vaccine efficacy using spores from the UK1 (BI/NAP1/027) strain. Hamsters were administered the high-dose spore challenge 20–21 weeks after single immunization. All of the PBS-immunized animals succumbed to spore challenge (Figure 3C). Strikingly, all of the SC-Ad6-TcdA/B vaccinated hamsters survived to the end of the study regardless of vaccine route. Comparison of the survival curves demonstrated that both i.n. and i.m. SC-Ad6-TcdA/B vaccinated animals had significantly better survival than PBS control animals. Weight loss was observed in all animals over the course of the experiment, but weight loss in SC-Ad6-TcdA/B animals had either stabilized or began to reverse by day 7 (unpublished observation). Given these obervations, we would expect these animals to survive beyond day 7 if they had not been euthanized.

In a separate experiment, groups of 10 hamsters were immunized a single time with 10^11^ virus particles of SC-Ad6-TcdA/B either i.n. or i.m. or with PBS. Blood was collected 3 or 4 days after immunization and tested using the same analyte panel as before. Similarly, no significant differences were observed between vaccinated and unvaccinated on either day 3 or 4 (Figure S4A,B). At day 4, CBCs were measured in half of the animals. There were increases in the percentage of neutrophils and a corresponding decrease in the percentage of lymphocytes in animals receiving vaccine, but the number of neutrophils and lymphocytes showed no differences (Figure S4C). i.m. immunized animals had increases in platelets, but these were still within normal ranges [39].

**Figure 4:**
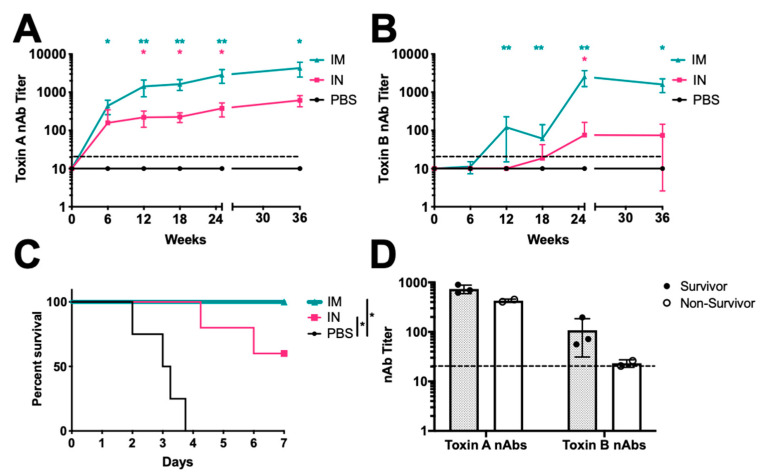
SC-Ad6-TcdA/B provides protection against lethal spore challenge 45 weeks after single immunization. Female Syrian hamsters (n = 10) were vaccinated i.n. or i.m. with 1 × 10^11^ virus particles of SC-Ad6-TcdA/B, or i.n. with PBS. Serum collected at weeks 6, 12, 18, 25, and 36 weeks after immunization were assayed to determine mean neutralizing titers for (**A**) toxin A and (**B**) toxin B. In both panels, points show the mean titer, bars show standard deviation, and the dotted line indicates the limit of detection (*Adjusted *p* < 0.05 compared to control, **Adjusted *p* < 0.05 compared to control and i.n. route). *X*-axis break represents the termination of the low-dose challenge study; serum collected at week 36 from remaining animals in each group (n = 5). (**C**) Survival curve for i.n. (n = 5) and i.m. (n = 4) SC-Ad-TcdA/B vaccinated animals challenged with UK1 spores 45 weeks after single immunization shows significant protection compared to PBS control animals (n = 4) (* *p* < 0.01). (**D**) Week 36 neutralizing toxin A and toxin B neutralizing titers of i.n. immunized animals that survived the challenge compared to non-survivors. Columns show the mean titer, bars show standard deviation, and the dotted line indicates the limit of detection.

Serum collected at weeks 6, 12, and 18 showed increasing TcdA and TcdB nAbs as in the first vaccination study (Figure 4A,B). At week 18, TcdA nAbs were over 160-fold greater than the controls in the i.m. group and 20-fold greater in the i.n. group. TcdB titers at this time were 6-fold greater in the i.m group and about 2-fold greater in the i.n group. At week 24, half of the hamsters in each group were challenged using the low-dose challenge method. This surprisingly induced no symptoms or indications of *C. difficile* infection in any hamster. Serum antibodies collected at their termination revealed no increases in TcdA or TcdB antibodies due to the challenge compared to unchallenged animals in the cohort. The unchallenged animals were followed for an additional 20 weeks. In this period, one PBS and one i.m. immunized animal became moribund and had to be euthanized at weeks 42 and 44, respectively.

The remaining animals were challenged at week 45 using the high-dose spore challenge (Figure 4C). All PBS immunized animals met endpoint criteria. Two intranasally immunized animals also succumbed. All animals in the i.m. group survived the challenge. Comparison of these survival curves showed significant differences in the survival of both i.n. and i.m. SC-Ad6-TcdA/B vaccinated animals compared to PBS. Toxin nAbs levels before challenge (week 36) correlated with survival in the groups (Figure 4D). Animals in the i.n. group that survived spore challenge had higher toxin nAbs prior to challenge compared to the those that did not survive. Importantly, protection against *C. difficile* challenge was observed 10 months after only a single immunization with the SC-Ad-TcdA/B vaccine.

**Figure 5:**
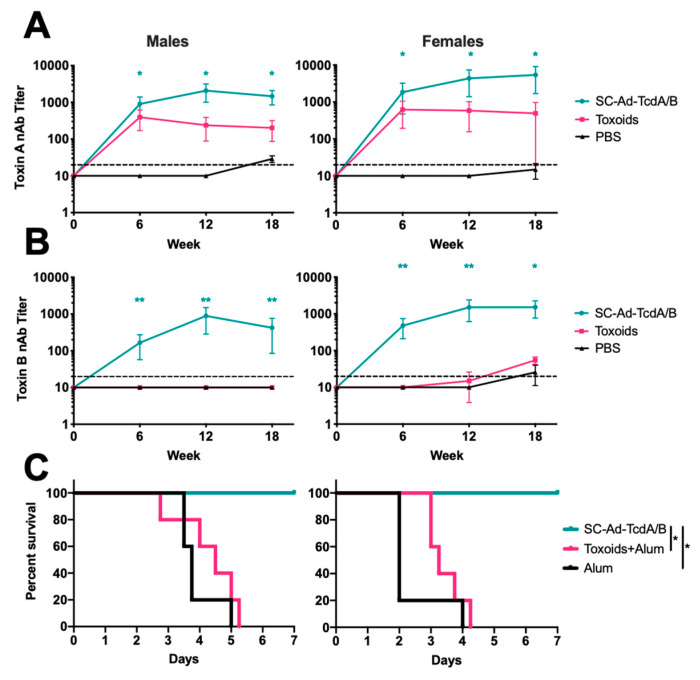
SC-Ad6-TcdA/B generates superior antibody and protection against lethal spore challenge compared to toxoid immunization. Male and female Syrian hamsters (n = 5) were vaccinated with SC-Ad6-TcdA/B, toxoids A and B, or Alum. Serum collected at 6, 12, and 18 weeks after immunization were assayed to determine mean neutralizing titers for (**A**) toxin A and (**B**) toxin B. In both panels, points show the mean titer, bars show standard deviation, and the dotted line indicates the limit of detection (* Adjusted *p* < 0.05 compared to control, ** Adjusted *p* < 0.05 compared to alum controls). (**C**) Survival curve for SC-Ad-TcdA/B vaccinated animals challenged with UK1 spores shows significant protection compared to toxoids A and B, and alum control animals (*p* < 0.01).

Groups of 5 male and 5 female Syrian hamsters were immunized once i.m. with 10^11^ virus particles of SC-Ad6-TcdA/B, 10 ug each toxoids A and B with alum, or alum alone. Serum collected at weeks 6, 12, and 18 showed increasing TcdA and TcdB nAbs in the SC-Ad6-TcdA/B (Figure 5A,B). In contrast, the toxoid immunization only generated TcdA antibodies. Twenty weeks after the single immunization, hamsters were challenged with the high dose of spores. In the males at week 18, TcdA nAbs were 140-fold greater than the controls in the SC-Ad6-TcdA/B immunized group and 20-fold greater in the toxoid immunized group, while the TcdB antibodies were 40-fold greater in the SC-Ad6-TcdA/B group and below the limit of detection in the toxoid group. In females, the TcdA nAb titers were over 350-fold and 30-fold greater than the controls in SC-Ad6-TcdA/B and toxoid groups, respectively. The TcdB nAb titers at the time were about 60-fold greater than the controls in the SC-Ad6-TcdA/B group and 2-fold greater in the toxoid group. All of the toxoid and alum immunized animals succumbed to spore challenge (Figure 5C). As in the pevious challenges, all of the SC-Ad6-TcdA/B vaccinated hamsters survived to the end of the study. Comparison of the survival curves demonstrated that toxoid immunized animals had no significant advantage compared to animals that only received alum. A single immunuzation with SC-Ad6-TcdA/B generated signifcant protection compared to both toxoid and alum immunized animals.

## 4. Discussion

In this study, we explored the utility of a replicating, single-cycle Ad (SC-Ad) vector as a *C. difficile* vaccine. We show that a single immunization of mice or hamsters with SC-Ad-TcdA/B generated rapid and robust antibody responses that persisted for more than 6 months. There were significantly higher antibody responses in female than in male mice. This bias is consistent with previous vaccine studies [43]. Despite these differences, single immunization afforded males and females with high titer, long-lasting toxin nAb responses that were protective of lethal TcdA challenge. 

Preventing mortality is seen as an important metric of vaccine efficacy in both mouse and hamster infection models of CDI. We challenged the mice with recombinant TcdA. In short-term and long-term vaccination studies, significant protection was afforded by SC-Ad6-TcdA/B against TcdA challenge. Mice that generated strong antibodies against the toxin survived, whereas those that failed to generate strong antibody levels did not. The mice that failed to generate a strong antibody response could have been a result of injection variation or immune system differences between the mice given they are outbred. Since these mice still generated immune responses, boosting the weak responders may improve their immune responses to provide protection.

A previous study by Seregin et al. created a replication-defective Ad vaccine against TcdA [34]. They tested its humoral and cellular immunogenicity in mice as well its utility in protecting mice against TcdA challenge. While we did not evaluate cellular immune responses in the present study, we suspect that our vaccine engaged this arm of the immunes system, as Ad vectors are known to drive robust T cell responses [44,45]. We evaluated antibody responses to TcdA and looked at protection in mice against TcdA challenge where we observed similar results to theirs in the mice that generated antibody responses to the vaccines were protected from lethal challenge. However, they challenged their mice at a much earlier time point (day 14) than in our studies (week 8 or week 38). Furthermore, we evaluated our vaccine in Syrian hamsters using the spore challenge, which is still consider the gold standard animal model of CDI [46]. Results in these studies suggest that both TcdA and TcdB responses are necessary for protection against CDI. Given this, we believe our vaccine have greater potential in preventing infections in humans.

Syrian hamsters were immunized once and challenged with the hypervirulent *C. difficile* strain BI/NAP1/027 (UK1) at different times after vaccination. Our vaccine was designed using toxin sequences from VPI 10463. Sequence comparisons of the strains’ toxins shows that the 3′ end of UK1′s TcdB is divergent. While the individual roles of the toxins during UK1 infection are unknown, analysis of mutant variants of another B1/NAP1/027 strain indicated that TcdA or TcdB alone are sufficient for virulence in hamsters [47]. If only TcdA were neutralized by our SC-Ad-TcdA/B vaccine, we would have expected our hamsters to have succumbed to infection as they did when they were vaccinated with the toxoids, which only generated TcdA nAbs. SC-Ad-TcdA/B immunization protected all i.m. immunized animals and most of the animals by the i.n. route with a subset of these animals succumbing at week 45. Vaccinated hamsters that survived *C. difficile* spore challenge had high toxin nAbs while those that succumbed had lower nAbs, which is consistent with our observations in mice. Thus, by extension, we believe that both toxins are sufficiently neutralized in our vaccinated hamsters during infection with the heterologous UK1 strain. 

The toxoid vaccines did not protect hamsters against challenge and were unable to significantly generate anti-Toxin B nAb responses. The hamsters intranasally immunized with SC-Ad-TcdA/B that did not survive challenge also had low levels of TcdB Ab titers. Together, these results indicate that it is likely that the ability to neutralize TcdB rather than TcdA has greater importance for protection. This is consistent with the approval of bezlotoxumab, which is a human monoclonal antibody that binds to TcdB. A Phase III trial showed bezlotoxumab to be beneficial in the prevention of recurrence of *C. difficile*-associated diarrhea, and these results led to its approval by the FDA [48,49]. The combination of actoxumab (an anti-TcdA antibody) and bezlotoxumab worked no better than bezlotoxumab alone [48]. This result was consistent with the indication that TcdB being the primary factor causing recurrent *C. difficile* infection in humans. However, it does not exclude TcdA as a contributing factor or indicate that anti-TcdA antibodies are protective in human disease, as is suggested by seroepidemiologic data [50]. 

While the use of Ads as vaccines vectors offer advantages, pre-existing immunity to Ads varies globally by Ad type [51], and pre-existing immunity can interfere with vaccine efficacy. The Ad serotype 5 (Ad5) is by far the most widely used adenovirus to be tested in humans in preclinical and clinical trials. This is unfortunate as most people already have immunity to Ad5 [52]. For this reason, we chose to use a lower seroprevalence Ad6 as it is as much as 50% less prevalent than Ad5 [53,54]. If pre-existing immunity to Ad6 proves to be a problem, it can be avoided through the use of rarer Ads, such as SC-Ad657, which was used as an HIV-1 vaccine [25]. 

Pre-existing Ad immunity can also be overcome by intranasal immunization [55,56,57]. It was for this reason, in part, that we tested i.n. immunization of our putative vaccine in our hamster study. Our other rationale for using this route was the hope that mucosal vaccination might mediate better protection against *C. difficile* in the mucosal tissues of the gut. However, these results suggest that i.n. immunization is not as robust as i.m. immunization for providing protective immunity against *C. difficile* when using the same dose. The results of our preliminary pharmacology and toxicology studies indicate that the dose of 10^11^ virus particles was well tolerated, so higher doses of the vaccine could be evaluated in subsequent studies if the i.n. route is desirable for ease of delivery or avoiding pre-existing Ad immunity.

## 5. Conclusions

In summary, the a single-cycle adenovirus targeting the receptor binding domains of both toxins A and B was tested in 2 separate mouse and 3 separate hamsters experiments. We show that this vaccine provides immunity and protection against *C. difficile* infection more than 10 months after a single immunization in both animal models. These data suggest that this SC-Ad *C. difficile* vaccine may have utility in humans. The ability to immunize once to achieve high titer antibodies against TcdA and TcdB and protection against clinically relevant strains of *C. difficile* may be a favorable feature for a vaccine. The SC-Ad vaccine may have use as a stand-alone vaccine or as a priming agent for protein vaccines that are currently in clinical testing.

## Supplementary Materials

The following are available online at https://www.mdpi.com/2076-393X/8/3/470/s1, **vaccines-891647**: Schematic of TcdA/B fusion protein and SC-Ad6 plasmids expressing fusion protein, Figure S2: Single-cycle adenovirus expression of *C. difficile* TcdA/B fusion protein, Figure S3: Blood chemistry of SC-Ad6-TcdA/B vaccinated animals, Figure S4: Blood chemistry and CBC of SC-Ad6-TcdA/B vaccinated animals, Table S1: Veterinary Hematology (CBC) Assay on the Abaxis VetScan HM5 Analyzer Results, Table S2 Blood Chemistry Assay on the Piccolo Xpress Chemistry Analyzer Results Table.
