# Supplementary material for: A Replicating Single-Cycle Adenovirus Vaccine Effective against Clostridium difficile
Source: Vaccines (Basel). 2020 Aug 22;8(3):470. doi: 10.3390/vaccines8030470 (PMC7564163; doi:10.3390/vaccines8030470)
Supplement: Supplementary file 1 [file vaccines-08-00470-s001.pdf]

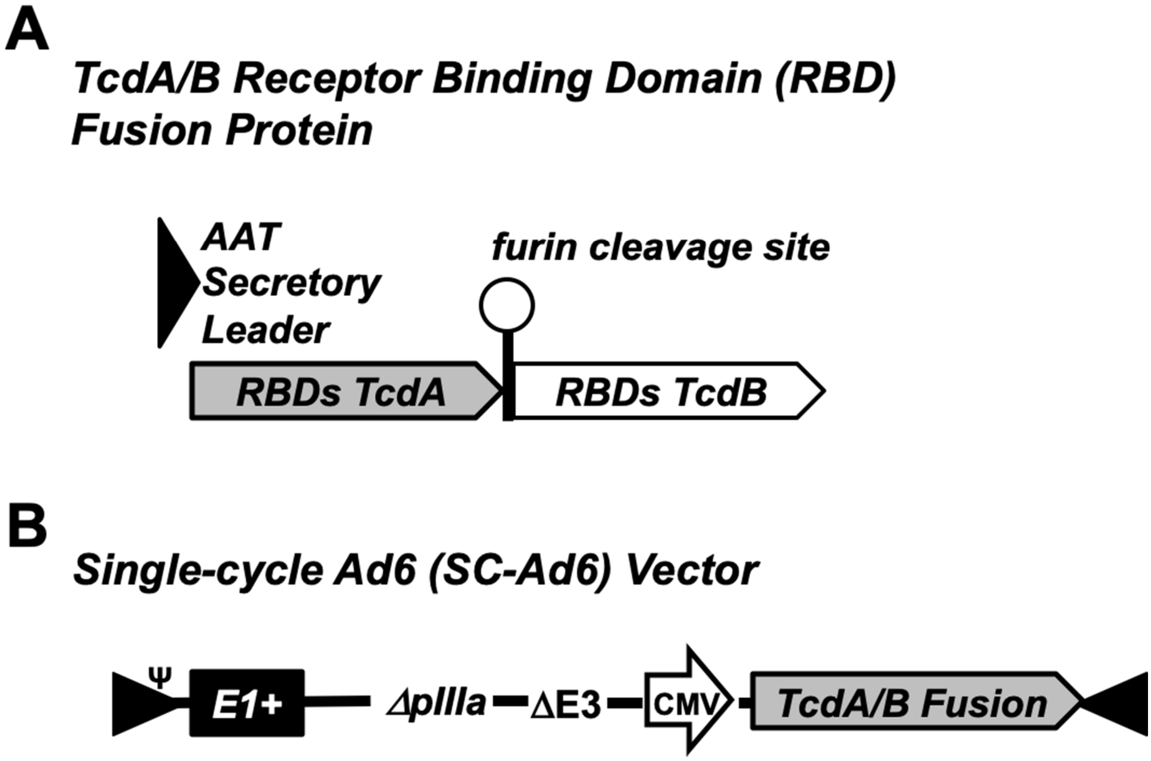

Supplemental Figure S1. Schematic of TcdA/B fusion protein and SC-Ad6 plasmids expressing fusion protein (A) TcdA/B fusion protein consists of a human *alpha* 1-antitrypsin (AAT) secretory leader sequence and the RBDs of TcdA and TcdB that are separated by two furin cleavage sites. **(B)** SC-Ad6, which is E1 expressing (E1+), protein IIIa deleted (ΔpIIIa), and E3 deleted (ΔE3) expressing the TcdA/B fusion protien using the CMV promoter.

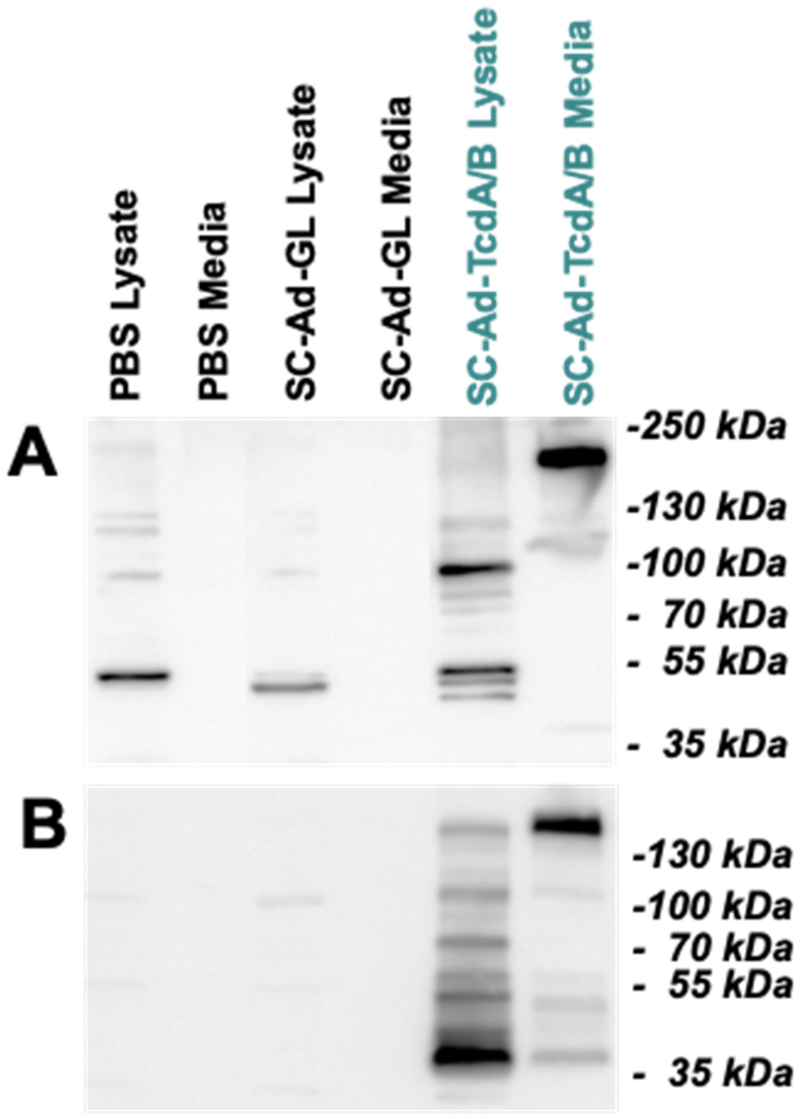

Supplemental Figure S2. Single-cycle adenovirus expression of *C. difficile* TcdA/B fusion protein. Western blot detecting expression of **(A)** TcdA and **(B)** TcdB fragments in the A549 cell lysates and media taken from uninfected cells, cells infected with an Ad control (SC-Ad6-GL), and cells infected with SC-Ad6-TcdA/B. The fusion protein (~MW 150 kDA), individual TcdA (~100 kDa) and TcdB (~50 kDa) fragments can be seen in SC-Ad6-TcdA/B infected cells. Non-specific binding to proteins the cells, and Ad control can be seen in both blots.

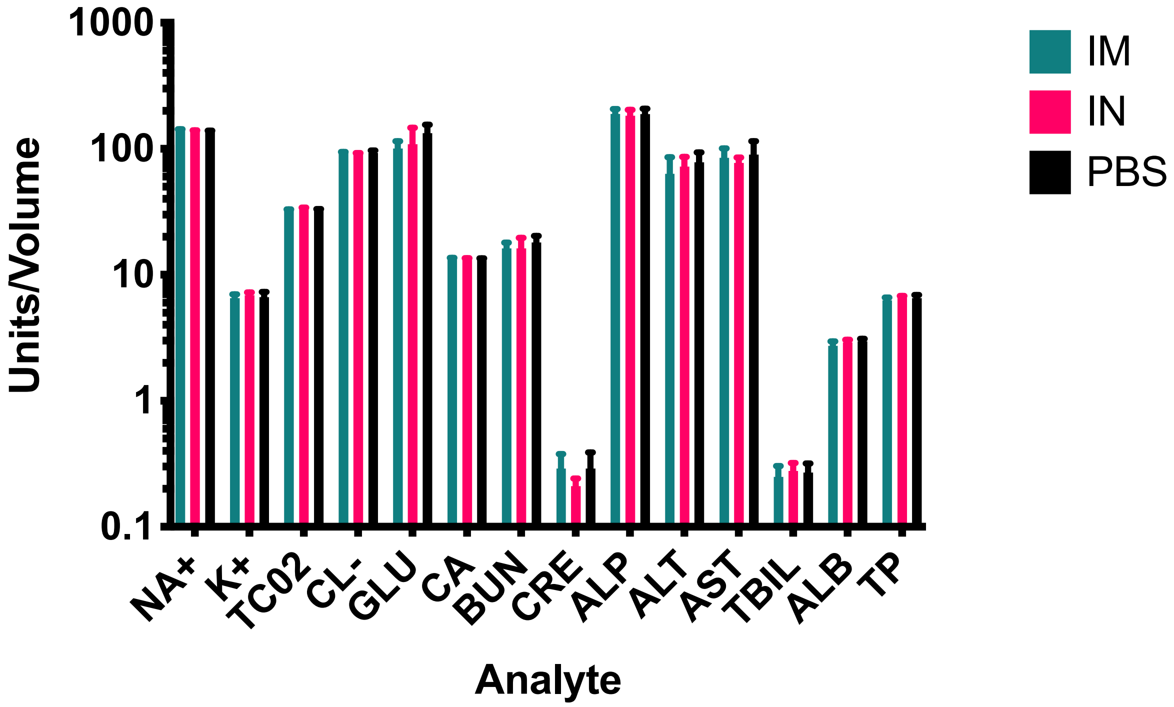

Supplemental Figure S3. Blood chemistry of SC-Ad6-TcdA/B vaccinated animals. Groups of 10 Syrian hamsters were immunized a single time with 10^{11} virus particles of SC-Ad6-TcdA/B by the i.n. or i.m. route. Control animals received i.n. PBS. Blood was collected 3 days after immunization for clinical chemistry. Abbreviations shown in Table S1

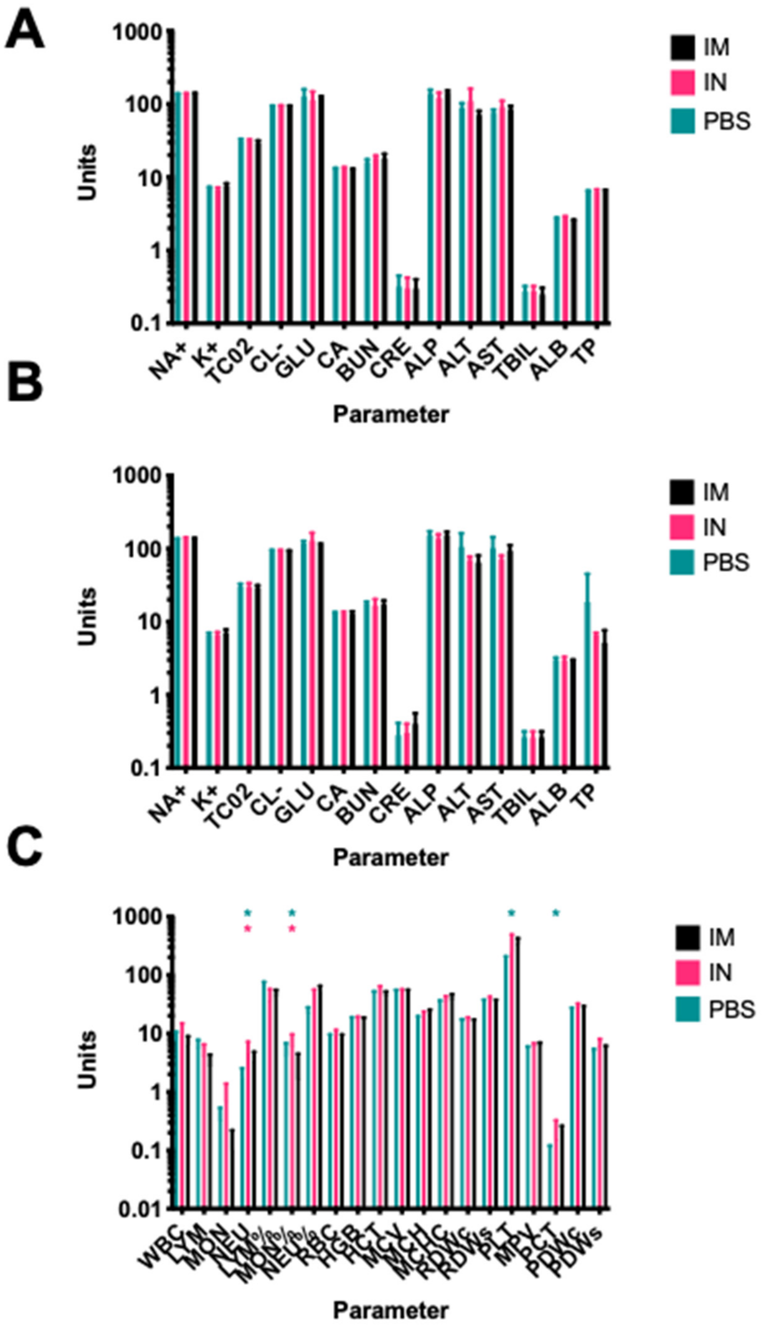

Supplemental Figure S4. Blood chemistry and CBC of SC-Ad6-TcdA/B vaccinated animals. Groups of 10 Syrian hamsters were immunized a single time with 10^{11}virus particles of SC-Ad6-TcdA/B by the i.n. or i.m. route. Control animals received i.n. PBS. Blood was collected ½ of the cohort (n=5 per group) **(A)** 3 and the other ½ of the cohort (n=5 per group) **(B)** 4 days after immunization for clinical chemistry. **(C)** Blood was collected on day 4 from ½ of the hamsters (n=5 per group) for CBC Abbreviations for panel A and B in Table S1 and in Table S2 for panel C.

Supplemental Table S1. Veterinary Hematology (CBC) Assay on the Abaxis VetScan HM5 Analyzer Results Table.

**Parameter**	**Abbreviation**	**Units**
Sodium	NA+	mmol/L
Potassium	K+	mmol/L
Total Carbon Dioxide	tCO2	mmol/L
Chloride	CL-	mmol/L
Glucose	GLU	mg/dL
Calcium	CA	mg/dL
Blood Urea Nitrogen	BUN	mg/dL
Creatinine	CRE	mg/dL
Alkaline Phosphatase	ALP	U/L
Alanine Aminotransferase	ALT	U/L
Aspartate Aminotransferase	AST	U/L
Total Bilirubin	TBIL	mg/dL
Albumin	ALB	g/dL
Total Protein	TP	g/dL

Supplemental Table S2. Blood Chemistry Assay on the Piccolo Xpress Chemistry Analyzer Results Table.

**Parameter**	**Abbreviation**	**Units**
Total White Blood Cell count	WBC	10^9L
Lymphocyte count	LYM	10^9L
Monocyte count	MON	10^9L
Neutrophil count	NEU	10^9L
Eosinophil count	EOS	10^9L
Basophil count	BAS	10^9L
Lymphocyte percentage	LYM%	%
Monocyte percentage	MON%	%
Neutrophil percentage	NEU%	%
Eosinophil percentage	EOS%	%
Basophil percentage	BAS%	%
Red Blood Cell count	RBC	10^12L
Hemoglobin	HGB	g/dL
Hematocrit percentage	HCT	%
Mean Corpuscular Volume	MCV	fL
Mean Corpuscular Hemoglobin	MCH	pg
Mean Corpuscular Hemoglobin Concentration	MCHC	g/dl
Red Cell Distribution Width, coefficient of variation %	RDWc	%
Red Cell Distribution Width	RDWs	fL
Platelet count	PLT	10^9L
Mean Platelet Volume	MPV	fL
Platelet crit %	PCT	%
Platelet Distribution Width, coefficient of variation %	PDWc	%
Platelet Distribution Width	PDWs	fL
